# Histopathological Changes Following Bromelain-Based Enzymatic Debridement (NexoBrid^®^): A Comprehensive Systematic Review of Preclinical and Clinical Evidence

**DOI:** 10.3390/medsci14010157

**Published:** 2026-03-23

**Authors:** Stefana Avadanei-Luca, Dan-Cristian Moraru, Andra-Irina Bulgaru-Iliescu, Raluca Tatar, Iulia Nacea, Alexandru Hristo Amarandei, Mihai-Codrin Constantinescu, Mihaela Pertea

**Affiliations:** 1 Grigore T. Popa University of Medicine and Pharmacy, 700115 Iasi, Romania; stefana_luca@umfiasi.ro (S.A.-L.); cristian-dan.moraru@umfiasi.ro (D.-C.M.); andra.bulgaru.iliescu@umfiasi.ro (A.-I.B.-I.); alexandru-hristo.amarandei@d.umfiasi.ro (A.H.A.); mihai-codrin.constantinescu@umfiasi.ro (M.-C.C.); mihaela.pertea@umfiasi.ro (M.P.); 2 Department of Plastic Surgery and Reconstructive Microsurgery, Sf. Spiridon Emergency County Hospital, 700111 Iasi, Romania; 3 Grigore Alexandrescu Clinica Emergency Hospital for Children Bucharest, 010621 Bucharest, Romania; iulia.nacea@umfcd.ro; 4 Department of Plastic Surgery, Carol Davila University of Medicine and Pharmacy, 020021 Bucharest, Romania

**Keywords:** NexoBrid^®^, bromelain, enzymatic debridement, burns, histology, histopathology, wound healing

## Abstract

**Background**: NexoBrid^®^ (NXB; MediWound Ltd., Yavne, Israel) (anacaulase-bcdb) is a bromelain-based enzymatic debriding agent approved for eschar removal in burn care. Despite widespread clinical use, histological evidence of tissue-level changes after enzymatic debridement remains limited. This systematic review aimed to evaluate preclinical and clinical studies describing histological findings following bromelain-based enzymatic debridement of thermal burns. **Methods**: Following PRISMA 2020 guidelines, we performed parallel systematic searches of preclinical (animal) and clinical (human) studies across PubMed, Embase, CENTRAL, Web of Science, and Scopus. Included studies reported thermal burns treated with bromelain-based enzymatic debridement and tissue biopsies with histological analysis. Quality was assessed using the SYRCLE Risk of Bias Tool (preclinical) and JBI Critical Appraisal Checklists (clinical). **Results**: Six preclinical studies (five porcine, one rat) met inclusion criteria. Findings included: selective eschar removal with dermal preservation; protection of the zone of stasis (67% partial- vs. 100% full-thickness necrosis; *p* = 0.05); viable dermal thickness of 1.1 ± 0.7 mm; and accelerated re-epithelialization (7.4 ± 0.8 vs. 9.1 ± 2.1 days; *p* < 0.05). Only two clinical studies (n *=* 9 patients) met the inclusion criteria: one case series (*n* = 8) and one case report. Clinical findings showed upper dermal homogenisation with preserved deep dermis, vascular congestion correlating with pinpoint bleeding, and pseudoeschar formation via transepidermal elimination. **Conclusions**: Preclinical evidence supports selective enzymatic debridement with dermal preservation. However, clinical histological data are limited to nine patients after over 13 years of use. This highlights a critical translational gap and underscores the need for prospective clinical histological studies.

## 1. Introduction

Burn injuries represent a substantial global health burden, with the World Health Organization estimating that approximately 11 million cases annually require medical attention [1]. Management of deep partial-thickness and full-thickness burns centres on timely eschar removal as a prerequisite for wound healing and infection prevention [2]. Surgical tangential excision has constituted the standard of care for over five decades, involving sequential sharp removal of tissue layers until viable dermis is encountered [3]. However, surgical debridement is inherently imprecise at the tissue level. The reliance on visual and tactile cues to distinguish viable from nonviable tissue results in variable amounts of healthy tissue sacrifice. This is particularly problematic in deep partial-thickness burns where the zone of stasis represents potentially salvageable tissue. Foundational histological research by Gurfinkel and colleagues demonstrated that surgical tangential excision sacrifices a mean of 41.2% viable tissue, with only 5.4% of excised samples containing exclusively necrotic tissue [4]. This finding established the critical need for more selective debridement approaches capable of discriminating between damaged and viable tissue at the molecular level.

NexoBrid^®^ (anacaulase-bcdb; MediWound Ltd., Yavne, Israel) represents a paradigm shift toward selective enzymatic debridement. This concentrate of proteolytic enzymes enriched in bromelain, derived from pineapple (Ananas comosus) stems, selectively degrades denatured extracellular matrix components within burn eschar while theoretically preserving viable underlying tissue [5]. The selectivity is predicated on differential substrate susceptibility: heat-denatured collagen undergoes conformational changes, rendering it vulnerable to proteolytic cleavage, whereas native collagen maintains structural resistance [6]. NexoBrid^®^ received European Medicines Agency marketing authorisation in December 2012 (EMEA/H/C/002246), with subsequent United States Food and Drug Administration approval in December 2022 for adults and August 2024 for paediatric patients [7,8]. Clinical efficacy has been established through multiple randomised controlled trials demonstrating superior debridement rates and reduced surgical intervention requirements [9,10,11].

It is essential to distinguish between true eschar and a superficial coagulum. True eschar is an irreversibly denatured protein matrix. It results from a full-thickness thermal injury. In these injuries, the temperature exceeds the threshold for complete protein coagulation through the entire thickness of the skin. In contrast, a superficial coagulum or dried exudate forms over partial-thickness wounds. It develops when plasma proteins and wound fluid dry on the surface of the wound. These represent fundamentally different substrates with distinct clinical implications. Enzymatic debridement acts on denatured proteins in both contexts but with different therapeutic objectives: in full-thickness burns, it removes the entire eschar layer to expose the underlying subcutaneous tissue or fascia, whereas in partial-thickness injuries, it removes the superficial damaged zone while theoretically preserving the underlying viable dermis capable of supporting re-epithelialisation. The term ‘pseudoeschar’ as used in this review refers specifically to the post-debridement phenomenon of transepidermal elimination described by Miura et al. [12]—a secondary formation occurring after enzymatic treatment that is distinct from both true eschar and pre-treatment wound exudate, and which represents physiological extrusion of damaged dermal components rather than residual or recurrent necrotic tissue.

While clinical outcome data establish treatment efficacy, histological evidence provides unique mechanistic insights. Understanding microscopic alterations induced by enzymatic debridement is critical for: (1) validating the selective debridement mechanism through direct tissue visualisation; (2) informing post-debridement wound care protocols; (3) optimising timing of subsequent interventions, particularly skin grafting; (4) characterising post-debridement phenomena such as pseudoeschar formation; and (5) enabling comparative analysis with alternative debridement modalities.

Preclinical studies using animal models have generated substantial histological data under controlled experimental conditions. Porcine skin shares anatomical and physiological similarities with human skin, making it a valuable translational model [13]. However, this preclinical evidence has not been systematically synthesised alongside the limited clinical histological data. This comprehensive review addresses this gap by examining both preclinical and clinical histological evidence in parallel arms.

This systematic review aims to: (1) identify all preclinical and clinical studies examining histological changes following bromelain-based enzymatic debridement of thermal burns; (2) critically appraise methodological quality using appropriate frameworks for each study type; (3) synthesise histological findings across tissue compartments and timepoints; (4) compare preclinical and clinical evidence to assess translational consistency; and (5) identify priorities for future research.

## 2. Materials and Methods

### 2.1. Protocol and Registration

This systematic review was conducted in accordance with the Preferred Reporting Items for Systematic Reviews and Meta-Analyses (PRISMA) 2020 statement [14]. The PRISMA 2020 checklist is provided as Supplementary Table S1. The clinical arm protocol was prospectively registered with PROSPERO (CRD420261278507). The preclinical arm was not registered, given PROSPERO’s scope limitation to human studies.

### 2.2. Eligibility Criteria

#### Preclinical Arm

Population: Animal models (porcine, rodent, or other species) with experimentally induced thermal burn injuries (flame, scald, contact burns). Non-thermal injury models (firearm wounds, chemical burns, ischaemic wounds) were excluded.

Intervention: Treatment with bromelain-based enzymatic debridement agents (NexoBrid^®^, Debrase^®^ [development-stage product name], or equivalent pineapple-derived proteases including ananain) (NXB; MediWound Ltd., Yavne, IsraelNXB; MediWound Ltd., Yavne, Israe). Studies using non-bromelain enzymatic agents (collagenase, papain) were excluded.

Outcome: Formal tissue biopsy with histological analysis including haematoxylin and eosin (H&E) staining, special stains, or immunohistochemistry. Studies with only visual or macroscopic wound assessment were excluded.

Study design: Controlled experimental studies with comparison groups.

### 2.3. Clinical Arm

Population: Human patients of any age with thermal burn injuries (flame, scald, contact, or flash burns). No restrictions were placed on burn depth, total body surface area (TBSA), anatomical location, or time since injury.

Intervention: Treatment with NexoBrid^®^ (anacaulase-bcdb) specifically. Studies using other enzymatic debriding agents were excluded to maintain specificity.

Outcome: Formal tissue biopsy with histological or histopathological analysis, defined as microscopy-based examination of tissue specimens obtained from burn wounds.

Study design: Any clinical study design including randomised controlled trials, cohort studies, case series, and case reports. Given the anticipated paucity of clinical histological evidence, case reports were deliberately included to ensure comprehensive capture of all available data, particularly for novel phenomena such as pseudoeschar formation that have not been characterised in larger studies. The limitations of case report evidence are explicitly acknowledged in our quality assessment and interpretation of findings.

### 2.4. Exclusion Criteria (Both Arms)

Studies were excluded if they met any of the following criteria: (1) non-thermal burn injuries (chemical, electrical, radiation, friction burns, firearm wounds, ischaemic wounds); (2) non-bromelain enzymatic agents; (3) studies without formal tissue biopsy/histological analysis; (4) reviews, meta-analyses, editorials, or commentaries without original data; (5) conference abstracts without full methodological details; (6) duplicate publications; and (7) ex vivo or in vitro laboratory studies without in vivo validation.

### 2.5. Information Sources and Search Strategy

A comprehensive literature search was conducted across PubMed/MEDLINE, Embase, Cochrane CENTRAL, Web of Science Core Collection, and Scopus. Clinical trial registries (ClinicalTrials.gov, EU Clinical Trials Register, WHO ICTRP) were searched for completed studies with histological outcomes. The search strategy combined controlled vocabulary and free-text terms: (NexoBrid^®^ OR anacaulase OR Debrase^®^ OR bromelain OR ananain) AND (burn OR thermal injury) AND (histology OR histopathology OR biopsy OR microscopy). For the preclinical arm, animal model terms were added. Database searches were conducted with no date or language restrictions up to 30 December 2025.

### 2.6. Study Selection and Data Extraction

Two reviewers independently screened titles and abstracts, followed by conducting a full-text review of potentially eligible studies. Disagreements were resolved by consensus. Data extraction captured study characteristics; population characteristics; intervention details; histological methods; and outcomes by tissue compartment and timepoint.

### 2.7. Quality Assessment

Preclinical studies were assessed using the SYRCLE Risk of Bias Tool for animal studies [15]. Clinical studies were assessed using Joanna Briggs Institute (JBI) Critical Appraisal Checklists [16]. Overall quality was categorised as high (≥75% “Yes”), moderate (50–74%), or low (<50%).

### 2.8. Data Synthesis

Given anticipated heterogeneity, narrative synthesis was employed following Synthesis Without Meta-analysis (SWiM) guidelines [17]. Certainty of evidence was assessed using a modified GRADE approach [18].

## 3. Results

### 3.1. Preclinical Evidence

#### 3.1.1. Study Selection

**Figure 1:**
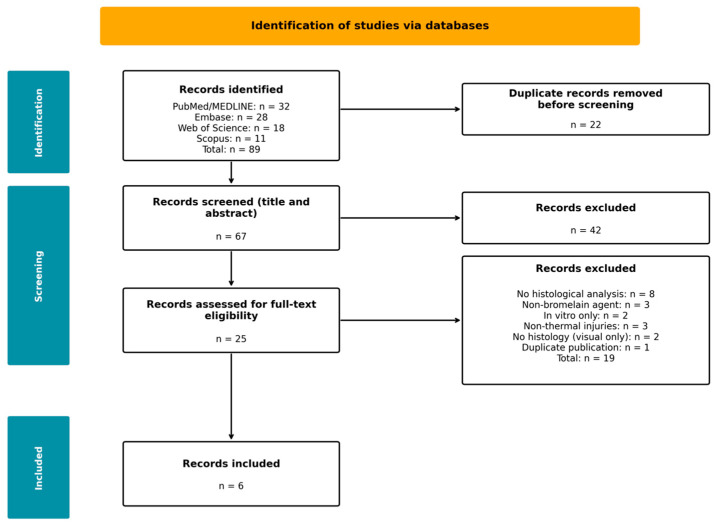
PRISMA 2020 flow diagram for the preclinical arm. Six studies met all inclusion criteria (five porcine models, one rat model).

The PRISMA flow diagram for the preclinical arm (Figure 1) summarises the study selection process. Database searching identified 89 records. After duplicate removal, 67 unique records underwent title/abstract screening, which excluded 42 records. Twenty-five full-text articles were assessed for eligibility. Full-text review excluded 19 records: eight lacked formal histological analysis; three used non-bromelain enzymatic agents; two were in vitro only; three involved non-thermal injuries (e.g., firearm wound models; an ischaemic wound model); two thermal burn studies lacked histological analysis and one was a duplicate publication. Six preclinical studies met all inclusion criteria: five porcine model studies (Orgill 1996 [19]; Singer 2010a [20]; Singer 2010b [21]; Singer 2011 [22]; Rosenberg 2012 [6]) and one rat model study (Rowan 1990 [23]). All included studies examined thermal burn injuries treated with bromelain-based enzymatic agents and performed formal histological analysis.

#### 3.1.2. Characteristics of Included Preclinical Studies

Table 1 presents the characteristics of the six included preclinical studies. All studies used bromelain-derived enzymatic debridement on thermal burns and conducted histological analyses (primarily H&E staining, with some employing special stains or morphometry). Burn models ranged from deep partial-thickness to full-thickness injuries. Sample sizes were modest (5–12 per study). Interventions included a purified pineapple stem protease (ananain) and bromelain-based formulations (Debrase^®^ or a bromelain gel dressing). Histological assessment time points varied from immediate post-debridement up to 14 days, capturing acute and early healing phases. Assessment timepoints ranged from immediate post-debridement evaluation (4–24 h in Rowan et al. [23] and Rosenberg et al. [6]) to extended follow-up through 14 days post-treatment (Singer et al. studies [20,21,22]). The differences in timing between studies prevented direct quantitative comparison. For this reason, we used a narrative synthesis instead of a meta-analysis. However, the studies still offered complementary evidence from different stages of wound healing. Acute-phase studies showed the immediate selectivity of enzymatic debridement. Studies with longer follow-up described the healing process over time. They also reported tissue-level outcomes, such as re-epithelialisation and dermal preservation.

#### 3.1.3. Quality Assessment

Quality assessment using the SYRCLE tool revealed moderate methodological quality overall for the animal studies. Singer et al. (2010a) [20] demonstrated the most rigorous methods, including randomisation and blinded histological outcome assessment. Common limitations across studies included unclear allocation concealment (reported in only one of six studies), absence of blinded outcome assessment (in four of six studies), and incomplete reporting of randomisation methods (in three of six studies). Nonetheless, all studies adequately described baseline burn characteristics and used appropriate statistical analyses.

#### 3.1.4. Preclinical Histological Findings

Selectivity of enzymatic debridement: The foundational selectivity study by Rosenberg et al. [6] systematically demonstrated that the bromelain-based debriding gel dressing selectively removed burn eschar while preserving viable tissue. Punch biopsies from partial-thickness burns, normal unburned skin, and skin graft donor sites revealed complete eschar dissolution in burn wounds, while normal skin and the donor site dermis remained histologically intact (Table 2).

Zone of stasis preservation: Singer et al. [20] provided quantitative histological evidence using a validated porcine comb burn model. At 48 h post-debridement, Debrase^®^-treated interspaces demonstrated significantly better outcomes: 67% showed partial-thickness necrosis versus 100% full-thickness necrosis in controls (*p* = 0.05). This finding provides direct histological evidence that enzymatic debridement preserves the zone of stasis.

Dermal thickness preservation: Histomorphometric analysis quantified viable dermal thickness following enzymatic debridement at 1.1 ± 0.7 mm [20]. Orgill et al. [19] demonstrated that enzymatically debrided wounds retained an acellular deeper dermis with a preserved collagen matrix structure.

Accelerated re-epithelialisation: Singer et al. [21] reported significantly faster complete re-epithelialisation: 7.4 ± 0.8 days for Debrase^®^-treated wounds versus 9.1 ± 2.1 days for controls (*p* < 0.05). At day 7, Debrase^®^-treated mid-dermal burns showed 47.6 ± 3.2% microscopic re-epithelialisation compared with 0% in untreated controls (*p* < 0.001) [22].

Collagen matrix architecture: Following enzymatic treatment, wound beds showed an acellular dermis with preserved collagen bundle organisation [19]. Rowan et al. [23] corroborated these findings in a rat model.

### 3.2. Clinical Evidence

#### 3.2.1. Study Selection

**Figure 2:**
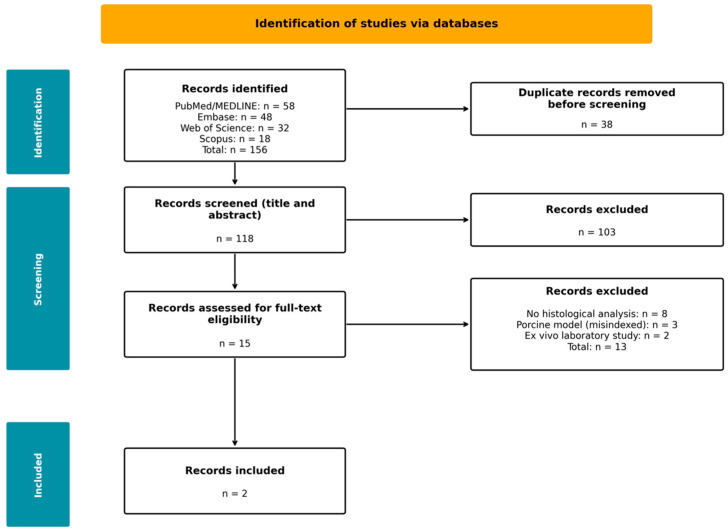
PRISMA 2020 flow diagram for the clinical arm. Two studies met all inclusion criteria (total n = 9 patients).

The PRISMA flow diagram for the clinical arm (Figure 2) summarises study selection. Database searching identified 156 records. After duplicate removal, 118 unique records underwent screening, excluding 103. Fifteen full-text articles were assessed for eligibility. Full-text review excluded 13 records: eight lacked formal histological analysis, three were porcine model studies incorrectly indexed, and two were ex vivo laboratory studies. Two studies met all inclusion criteria.

#### 3.2.2. Characteristics of Included Clinical Studies

Only two studies met the inclusion criteria for the clinical arm: Di Lonardo et al. (2018), a prospective case series from Italy [24], and Miura et al. (2025), a case report from Japan [12]. Table 3 presents detailed study characteristics. Briefly, Di Lonardo et al. included eight adult patients (mean age ~49 years, five male/three female) with mid-to-deep partial-thickness burns (average ~30% TBSA) treated with NexoBrid^®^ within 24 h of injury. Miura et al. [12] reported on a single male patient (50 s, ~6% TBSA flame burn) treated with NexoBrid^®^ ~22 h post-injury. The primary objective in Di Lonardo et al. [24] was to characterise pre- and post-debridement histology, whereas Miura et al. [12] focused on the histopathology of the post-debridement pseudoeschar phenomenon.

#### 3.2.3. Biopsy Protocols and Histological Methods

Table 4 details biopsy protocols. Di Lonardo et al. [24] obtained paired pre- and post-debridement biopsies using the 4 mm punch technique with immediate formalin fixation. Miura et al. [12] obtained a single biopsy at day 10 post-injury to characterise pseudoeschar. Neither study employed immunohistochemistry. Punch biopsies (4 mm) were employed as the standard technique due to their minimal invasiveness and adequate tissue depth for histological evaluation of all dermal layers. Excisional biopsies (~3 cm) were reserved for select cases where larger tissue samples were clinically indicated to evaluate broader wound bed characteristics or transition zones and where anatomical location permitted more extensive sampling without compromising wound healing or functional outcomes.

#### 3.2.4. Quality Assessment

Di Lonardo et al. [24] achieved moderate quality (6/8 items, 75%) with limitations including unclear consecutive patient enrolment. Miura et al. [12] achieved moderate quality (5/8 items, 62.5%) with incomplete demographic reporting and absence of specified burn depth, which limited correlation of histological findings with injury severity and precluded assessment of depth-specific outcomes.

#### 3.2.5. Clinical Histological Findings

Pre-debridement histopathology: Pre-debridement biopsies (n = 8) demonstrated characteristic deep partial-thickness burn pathology: complete epidermal absence, unevenly distributed coagulative necrosis, polymorphonuclear neutrophil (PMN)-predominant inflammatory infiltrates, and partially preserved adnexa with cytologic alterations including pyknotic nuclei with perinuclear haloes.

The homogenisation phenomenon: The central clinical histological finding was post-debridement upper dermal “homogenisation.” The superficial (papillary) dermis exhibited a distinctive homogenised, matrix-like appearance qualitatively different from normal dermis. Di Lonardo et al. [24] characterised this layer as functionally analogous to synthetic dermal matrix scaffolds, capable of supporting tissue regeneration when maintained in an appropriately moist environment. Critically, the deep (reticular) dermis preserved normal morphological features with intact structural integrity. This differential effect—superficial homogenisation with deep preservation—provides histological evidence consistent with selective enzymatic action.

Vascular changes: Post-debridement biopsies revealed prominent vascular congestion with capillary disruption and blood extravasation. These findings correlate directly with the clinical observation of pinpoint bleeding following NexoBrid^®^ removal.

Pseudoeschar formation: Miura et al. [12] provided the first detailed histopathological characterisation of pseudoeschar. Histological examination revealed a composition of primarily collagen and elastic fibres, with inflammatory cells, collagen, and elastic fibres ascending from the lower dermis through the ulcer layer. The authors proposed that pseudoeschar formation involves transepithelial elimination of degenerated dermal components, analogous to perforating dermatoses.

This characterisation has immediate clinical significance: pseudoeschar represents physiological extrusion rather than treatment failure, supporting conservative management.

The interface layer: Both sets of authors stressed the importance of managing the wound bed left after enzymatic debridement. Di Lonardo et al. [24] in particular highlighted that the homogenised dermal layer (interface layer) must be kept in an optimal environment. If allowed to dry out (desiccate), this proteinaceous matrix could harden into a “neo-eschar” that might impede healing. Therefore, moist wound healing techniques are recommended post-NexoBrid^®^ to preserve the viability and functionality of this dermal scaffold. This aligns with European consensus guidelines that advise specific post-enzymatic debridement wound care practices [25,26]. All these results are included in Table 5.

## 4. Discussion

This systematic review uncovered a clear disparity between the volume of preclinical and clinical histological evidence for bromelain-based enzymatic debridement. The preclinical arm yielded six studies in animal models, providing robust and often quantitative histological data. In contrast, the clinical arm yielded only two studies, encompassing a total of nine patients. This translational gap is a primary finding of the review [25,26]. Despite over a decade of clinical NexoBrid^®^ use worldwide, detailed human histological data remain exceedingly scarce.

Integration of preclinical and clinical evidence:

Despite this quantitative disparity, the evidence demonstrates remarkable consistency across domains.

### 4.1. Deep Dermal Preservation

The post-debridement “homogenisation” phenomenon observed in human tissue (Di Lonardo et al. [24]) parallels the histomorphometric findings in porcine studies (Singer et al. 2010a [20]). In both humans and pigs, enzymatic debridement modifies or removes the superficial necrotic layer while leaving the deeper dermis structurally intact. Singer et al. quantified viable dermis ~1.1 mm thick in pigs [20]; although no exact measurement exists in humans, the qualitative descriptions suggest a comparable preservation of dermal depth.

Selective action on damaged vs. healthy tissue:

Rosenberg et al.’s porcine selectivity study showed that enzymatic treatment digested burn eschar yet left normal skin unharmed [6]. The clinical correlate is Di Lonardo et al.’s [24] observation that only the superficial burn layer was affected while the underlying viable dermis retained normal histology. Together, these support the proposed mechanism of differential collagen susceptibility (denatured vs. native) and underscore that bromelain’s effects are confined to the intended targets.

### 4.2. Zone of Stasis Protection

Singer et al.’s porcine data (67% vs. 100% necrosis in zone of stasis regions) provide strong evidence that enzymatic debridement preserves marginally viable tissue [20]. Clinically, we infer similar protection: the fact that debrided human burns showed viable dermis and vascular patency suggests that tissue which might have otherwise progressed to necrosis (if left or excised surgically) was salvaged. While controlled human data are lacking, the preservation of dermal architecture in NexoBrid^®^-treated patients is consistent with protecting the zone of stasis in burns.

### 4.3. Pseudoeschar Phenomenon

Miura et al.’s [12] description of pseudoeschar formation has no direct analogue in the animal studies. The pseudoeschar phenomenon as characterised by Miura et al. represents a single-case observation that, while providing a plausible mechanistic explanation for clinical observations, cannot be considered definitive or generalisable. The proposed mechanism of transepidermal elimination of degenerated dermal components is biologically plausible and consistent with similar phenomena observed in perforating dermatoses, but this histopathological characterisation requires independent validation in prospective studies with larger sample sizes before it can inform clinical management decisions. Until such validation is available, clinicians should interpret pseudoeschar as a phenomenon warranting further investigation rather than a definitively characterised entity.

### 4.4. Mechanistic Synthesis

The collective evidence supports a unified mechanistic model [5,6]:

(1) Collagen conformational selectivity: The native collagen triple-helix structure resists proteolysis, while heat-denatured collagen (>60 °C) becomes susceptible to bromelain proteases [27]. (2) α_2_-Macroglobulin protection: This endogenous protease inhibitor in viable tissue provides additional protection against enzymatic degradation. (3) Depth-dependent temperature gradient: Thermal energy attenuation with depth creates natural selectivity. (4) Cell viability preservation: Bromelain demonstrates >70% cell viability at therapeutic concentrations in vitro [28].

### 4.5. Clinical Implications

Burn depth and grafting strategy considerations: The tissue-sparing benefits of enzymatic debridement must be interpreted within specific clinical contexts rather than as a universal advantage. For full-thickness burns, where a viable dermis is absent by definition, tissue preservation is not a relevant endpoint—the primary therapeutic objective is achieving a well-vascularised wound bed (typically subcutaneous fat or fascia) capable of supporting graft survival, and enzymatic debridement should be evaluated on the criteria of debridement completeness, time efficiency, and wound bed quality rather than tissue preservation per se. For superficial and mid-partial-thickness burns, which typically heal spontaneously through re-epithelialisation from surviving adnexal structures and wound margins, the role of enzymatic debridement is to accelerate natural eschar separation rather than replace surgical intervention. For deep partial-thickness burns, the clinical relevance of tissue preservation depends critically on the planned reconstruction strategy. When sheet grafting is intended for optimal cosmesis (e.g., face, hands, and visible joints), complete removal of viable dermis may actually be preferable to prevent inclusion cyst formation from residual epithelial appendages trapped beneath the graft—a complication that can compromise both cosmetic and functional outcomes and may require secondary surgical intervention. In these scenarios, the tissue-preserving properties of enzymatic debridement could theoretically be disadvantageous, though this specific question has not been systematically studied. Conversely, when mesh grafting or secondary healing is planned, preservation of the dermal matrix scaffold provides clear advantages for wound bed vascularity, reduced wound contraction, and potentially superior long-term scarring outcomes. Future research should stratify outcomes by burn depth and planned reconstruction approach to better define the clinical contexts in which tissue preservation translates to meaningful patient benefit.

Post-debridement wound care: The homogenised superficial dermis left by enzymatic debridement is essentially a biologic scaffold but is also fragile. It must be kept moist and protected. If it dries and forms a scab (a “neo-eschar”), it could impede healing or even necessitate additional debridement. Current European consensus guidelines already emphasise moist wound healing after NexoBrid^®^ [25,26], a recommendation strongly supported by these histological findings.

Optimal timing of skin grafting: Many burn centres wait ~2 days after enzymatic debridement before autografting. Histologically, this delay likely allows the homogenised dermal layer to stabilise and revascularise to some extent. Our findings support this practice: grafting too early could risk placing skin grafts on an unstable matrix, whereas waiting a short period may improve graft take by allowing the wound bed to “declare” itself and any pseudoeschar to separate.

Pseudoeschar management: Clinicians observing a pseudoeschar might worry that necrotic tissue has re-formed or that the initial debridement is incomplete. The histology shows otherwise: pseudoeschar is composed of expelled matrix elements, not newly necrosed tissue. Therefore, rather than reapplying debridement or surgically excising the pseudoeschar, a conservative approach (e.g., dressings to aid natural separation) is warranted. This avoids unnecessary trauma to the healing wound. However, the pseudoeschar findings derive from a single case report (n = 1) and should therefore be interpreted as preliminary and hypothesis-generating rather than definitive or generalisable. These observations require prospective validation in larger, diverse patient cohorts before firm clinical recommendations can be made.

Comparison to surgical tangential excision: Although we lack a direct head-to-head histological comparison in humans, the data suggest enzymatic debridement is much more tissue-sparing. Gurfinkel et al. [4] found that surgical excision removes significant viable dermis (often >40%). The enzymatic approach, by contrast, preserves the dermis and even some adnexal elements. In practical terms, this could mean better functional outcomes (less contraction, better graft beds) and perhaps improved scarring, though long-term outcomes were not specifically evaluated here.

### 4.6. Comparison with Surgical Debridement *(Indirect Evidence)*

**Figure 3:**
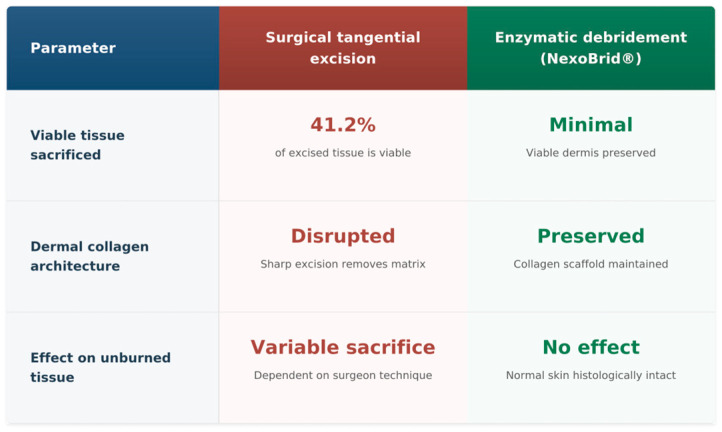
Comparison of histological outcomes between surgical tangential excision and bromelain-based enzymatic debridement. Surgical excision data are derived from Gurfinkel et al. [4], demonstrating that 41.2% of surgically excised tissue is viable. Enzymatic debridement selectivity data are derived from Rosenberg et al. [6] and Orgill et al. [19].

Gurfinkel et al.’s foundational study [4] demonstrated that surgical tangential excision sacrifices approximately 41.2% of viable tissue, with only 5.4% of excised samples containing exclusively necrotic material. This establishes an important quantitative benchmark for tissue sacrifice with conventional surgical approaches. While direct controlled comparison with enzymatic debridement is lacking, the quantitative zone of stasis data from Singer et al. [20,21,22] (67% partial-thickness vs. 100% full-thickness necrosis in untreated controls; *p* = 0.05) suggests potential for greater tissue preservation with enzymatic approaches. However, it is essential to acknowledge that these data derive from entirely different studies conducted in different species (human vs. porcine), with different burn models, assessment methods, and timepoints. Furthermore, as discussed above, tissue preservation is not uniformly desirable across all clinical scenarios—its relevance depends on burn depth and planned reconstruction strategy. Therefore, a definitive conclusion regarding the superiority of enzymatic over surgical debridement for tissue preservation cannot be drawn from the available evidence. Prospective controlled trials directly comparing the two modalities with histological endpoints in matched patient populations, stratified by burn depth and grafting approach, are required to resolve this question [Figure 3].

### 4.7. Limitations

Preclinical limitations: Species differences despite anatomical similarities; controlled laboratory conditions differing from clinical heterogeneity; predominantly short-term follow-up; three of six preclinical studies (Singer et al. 2010a [20], 2010b [21], 2011 [22]) originated from the same research group at Stony Brook University, potentially introducing consistency bias from shared experimental protocols, animal handling procedures, burn induction methods, and histological assessment techniques, while also limiting independent replication of findings by other laboratories; and two preclinical studies (Orgill et al. 1996 [19], Rosenberg et al. 2012 [6]) did not report sample sizes, limiting quantitative interpretation of their findings and precluding formal assessment of statistical adequacy.

Clinical limitations: Extremely small sample sizes (nine patients total); qualitative rather than quantitative analysis; limited temporal sampling; absence of controlled comparisons; geographic limitation to single centres; and incomplete methodological reporting in clinical studies, including unspecified tissue fixation protocols in Miura et al. [12] and absence of specified burn depth, limited reproducibility assessment and correlation of findings with injury severity.

Review limitations: Evidence scarcity (eight studies total); the preclinical arm protocol was not prospectively registered; potential publication bias; and the search may have missed non-English literature.

### 4.8. Future Research Priorities

This review highlights a clear need for further research, particularly on the clinical side:

1. Prospective clinical studies with systematic biopsies: We recommend new studies in burn patients where biopsies are taken at standardised intervals—e.g., immediately after debridement, 48–72 h post-debridement, 1–2 weeks post-debridement, and at healing. This would map the histological progression, including pseudoeschar dynamics and re-epithelialisation, providing much richer data.
2. Quantitative histological analyses: Future studies should incorporate histomorphometry (e.g., measuring depth of residual dermis and area of re-epithelialisation) and perhaps new imaging modalities.
3. Immunohistochemistry and molecular assays: Adding IHC for markers of interest (proliferation markers like Ki-67 to gauge regenerative activity, inflammatory markers like CD68 for macrophages, vascular markers like CD31/CD34 to assess neovascularisation, collagen subtyping for matrix changes, etc.) would dramatically enhance the understanding of how enzymatic debridement affects the wound biology beyond basic H&E morphology.
4. Controlled comparisons with surgical debridement within the same patient where clinically appropriate.
5. Correlation with long-term scar quality outcomes.

In summary, bridging the translational gap will require focused clinical research to build on the mechanistic foundation established by preclinical studies.

## 5. Conclusions

In this systematic review, we identified six preclinical studies demonstrating consistent histological benefits of bromelain-based enzymatic burn debridement: preservation of the burn zone of stasis (67% partial- vs. 100% full-thickness necrosis; *p* = 0.05), retention of viable dermal thickness (~1.1 ± 0.7 mm of dermis post-debridement), accelerated re-epithelialisation (7.4 ± 0.8 vs. 9.1 ± 2.1 days to heal; *p* < 0.05), and maintenance of the dermal collagen matrix architecture after eschar removal.

Clinical histopathological evidence, however, remains limited to two studies (nine patients total) despite NexoBrid^®^’s widespread global use and over 13 years of regulatory approval. The available human data show post-debridement upper dermal homogenisation with preserved deep dermis—findings that mirror the preclinical results—as well as vascular congestion corresponding to the pinpoint bleeding clinical sign, and the unique observation of pseudoeschar formation via transepithelial elimination of residual dermal components. The histopathological characterisation of pseudoeschar, while providing novel mechanistic insight into a commonly observed clinical phenomenon, derives from a single case and requires validation in larger prospective studies with systematic biopsy protocols before it can reliably inform clinical practice or wound management decisions.

This critical translational gap—the primary finding of our review—underscores an urgent need for prospective clinical histological studies of enzymatic debridement. Prospective clinical histological studies with quantitative histomorphometry, immunohistochemical analyses, and direct comparison to surgical debridement are urgently needed to validate these tissue-level benefits and optimise post-debridement wound care protocols.

## Figures and Tables

**Table 1:** Characteristics of included preclinical studies with histological outcomes.

Study (Year)	Model	Burn Type	Enzymatic Agent	n	Histology Methods	Timepoints	Key Histological Findings
**Rowan 1990** [23]	Rat	Full-thickness contact burn	Pineapple stem enzyme fractions	12	H&E staining	4 h, 24 h post-treatment	Rapid eschar removal within 4 h; selective eschar removal with preservation of viable tissue
**Orgill 1996** [19]	Porcine	Full-thickness contact burn	Ananain (purified pineapple cysteine protease)	NR	H&E; histomorphometry	Post-debridement	Acellular dermis with preserved collagen matrix; necrotic debris removed; dermal scaffold structure maintained
**Singer 2010a** [20]	Porcine	Comb burns (alternating partial- and full-thickness)	Debrase (bromelain)	6	H&E; blinded histological assessment	Days 1, 2, 3, 7	Zone of stasis preservation: 67% partial vs. 100% full-thickness necrosis (*p* = 0.05); viable dermal thickness 1.1 ± 0.7 mm
**Singer 2010b** [21]	Porcine	Deep partial-thickness burn	Debrase (bromelain)	6	H&E; re-epithelialisation assessment	Days 3, 5, 7, 10, 14	Accelerated healing: complete re-epithelialisation 7.4 ± 0.8 vs. 9.1 ± 2.1 days (*p* < 0.05); preserved dermal architecture
**Singer 2011** [22]	Porcine	Mid-dermal burns	Debrase (bromelain)	5	H&E; epithelialisation percentage	Days 3, 5, 7, 10, 14	Day 7 epithelialisation: 47.6 ± 3.2% vs. 0% in controls (*p* < 0.001); selective debridement with no damage to viable tissue
**Rosenberg 2012** [6]	Porcine	Partial-thickness burns plus normal skin and donor sites	Debriding gel dressing (bromelain-based)	NR	H&E; punch biopsies	Pre- and post-4 h application	Selectivity confirmed: complete burn eschar dissolution; normal skin and donor site dermis histologically unaffected

H&E = haematoxylin and eosin; n = number; DGD = debriding gel dressing; NR = not reported. Absence of sample size reporting in Orgill et al. (1996) [19] and Rosenberg et al. (2012) [6] limits statistical interpretation, precludes assessment of study power, and conflicts with contemporary standards for animal research reporting (ARRIVE guidelines). Quantitative findings from these studies should be interpreted with appropriate caution.

**Table 2:** Key quantitative histological outcomes from preclinical studies.

Outcome Measure (Study)	Enzymatic Debridement Result	Control/Comparison Result	*p*-Value
**Zone of stasis necrosis** (Singer 2010a [20])	67% partial-thickness injury	100% full-thickness injury	0.05
**Viable dermal thickness** (Singer 2010a [20])	1.1 ± 0.7 mm (preserved dermis)	0 mm (all necrotic)	— *(NA)*
**Complete re-epithelialisation time** (Singer 2010b [21])	7.4 ± 0.8 days	9.1 ± 2.1 days	<0.05
**Day 7 re-epithelialisation %** (Singer 2011 [22])	47.6 ± 3.2%	0%	<0.001
**Effect on normal skin** (Rosenberg 2012 [6])	No histological change (intact)	– *(normal skin not treated)*	— *(NA)*

(NA = not applicable; comparisons not available or not relevant).

**Table 3:** Characteristics of included clinical studies.

Characteristic	Di Lonardo et al., 2018 [24] (Case Series)	Miura et al., 2025 [12] (Case Report)
**Study Design**	Prospective case series	Case report
**Sample Size**	Eight patients	One patient
**Patient Age**	Mean 49 years	Male, 50 y
**Sex (M/F)**	Five M/three F	One M/zero F
**Burn Aetiology**	Not reported (mixed causes)	Flame
**Burn Depth**	Mid-deep to deep partial thickness	Not specified
**TBSA Burned**	~30% (mean)	6%
**Main Anatomical Location**	Lower limbs (biopsy sites)	Right shoulder and upper arm
**Time to NexoBrid^®^**	<24 h post-injury	~22 h post-injury
**Primary Objective**	Characterise pre- and post-debridement histology	Characterise pseudoeschar histopathology

TBSA = total body surface area; M = male; F = female.

**Table 4:** Biopsy protocols and histological methods in clinical studies.

Parameter	Di Lonardo et al., 2018 [24]	Miura et al., 2025 [12]
**Total Biopsies**	18 biopsies	One biopsy
**Pre-debridement Biopsies**	Eight (immediately before NexoBrid^®^)	None
**Post-debridement Biopsies**	Ten (immediately after NexoBrid^®^ removal; in some cases also ~24 h post-debridement)	One (day 10 post-injury, i.e., nine days after NexoBrid^®^)
**Biopsy Technique**	4 mm punch biopsies; ~3 cm excisional skin samples in select cases	Standard excisional biopsy (technique NR)
**Tissue Fixation**	Immediate fixation in 10% formalin	Not reported
**Staining**	Haematoxylin and eosin (H&E)	H&E (routine histopathology)
**Microscopy**	Light microscopy (multiple magnifications up to 40×)	Light microscopy (magnification not reported)
**Analysis Approach**	Qualitative descriptive histopathology	Qualitative descriptive histopathology
**Immunohistochemistry**	Not performed	Not performed

H&E = haematoxylin and eosin.

**Table 5:** Comparison of clinical histological findings by tissue compartment (pre- vs. post-debridement and pseudoeschar).

Tissue Compartment	Pre-Debridement (Di Lonardo 2018 [24])	Post-Debridement (Di Lonardo 2018 [24])	Pseudoeschar (Miura 2025 [12])
**Epidermis**	Complete loss of epidermis; no viable keratinocytes (full-thickness epidermal necrosis)	Absent (no re-epithelialisation immediately post-debridement); basement membrane status not reported	Ulcerated surface (open wound); pseudoeschar lies above the wound bed (extruded material)
**Superficial (Papillary) Dermis**	Coagulative necrosis of upper dermis; collagen denaturation and structural loss in focal areas	“Homogenised” appearance—acellular matrix-like layer serving as a dermal scaffold; capable of supporting regeneration under moist conditions	Source of pseudoeschar material—degenerated collagen and elastic fibres are extruded from here via transepithelial elimination
**Deep** **(Reticular)** **Dermis**	Partially preserved depending on burn depth; some viable structures may remain	Preserved normal morphology—intact collagen bundle organisation; viable tissue architecture maintained; increased inflammatory cell presence	Origin of ascending fibres and cells—inflammatory cells and connective tissue fragments travel from here to the surface, contributing to pseudoeschar
**Skin Adnexa (Glands,** **Follicles)**	Partially present but damaged; pyknotic nuclei and other signs of thermal injury in remaining adnexal cells	Greatly reduced in number; only a few appendage remnants observable (mostly in deeper dermis); limited viability	Not specifically described (not a focus of pseudoeschar composition)
**Vasculature**	Microvascular damage; thrombosis in severely burned areas; stagnant blood in zone of stasis	Significant vascular congestion in superficial dermis; capillary disruption with blood extravasation (pinpoint bleeding correlates)	Not described in detail (pseudoeschar primarily composed of connective tissue, not vasculature)
**Inflammatory Infiltrate**	Predominantly neutrophils (PMNs) throughout the injured dermis, both interstitial and perivascular	Neutrophil-predominant infiltrate persists; acute inflammation is ongoing post-debridement (part of normal wound healing cascade)	Inflammatory cells (including neutrophils and macrophages) found within pseudoeschar, migrating upward from dermis to surface
**Extracellular Matrix**	Burn eschar with denatured collagen; loss of normal dermal architecture in necrotic zones	Homogenised matrix in superficial dermis; normal collagen structure preserved in deep dermis	Collagen and elastic fibres present in pseudoeschar; these originate from the dermis and have been expelled to the surface
**Overall** **Interpretation**	Typical deep partial-thickness burn histology: mixed viable and necrotic tissue; evidence of a zone of stasis	Selective debridement achieved: necrotic superficial tissue removed or transformed; viable deep tissue spared; wound bed left with a regeneration-capable scaffold	Transepithelial elimination: pseudoeschar represents normal elimination of debris, not treatment failure; should be managed conservatively (part of healing process)

## Data Availability

No new data were created or analysed in this study.

## Supplementary Materials

The following supporting information can be downloaded at: https://www.mdpi.com/article/10.3390/medsci14010157/s1, The PRISMA 2020 checklist is provided as Supplementary Table S1.
