# Supplementary material for: Histopathological Changes Following Bromelain-Based Enzymatic Debridement (NexoBrid®): A Comprehensive Systematic Review of Preclinical and Clinical Evidence
Source: Med Sci (Basel). 2026 Mar 23;14(1):157. doi: 10.3390/medsci14010157 (PMC13028588; doi:10.3390/medsci14010157)
Supplement: Supplementary file 1 [file medsci-14-00157-s001.zip › medsci-4167152-supplementary.pdf]

PRISMA 2020 Checklist

Section and Topic	Item #	Checklist item	Location where item is reported
**TITLE**			
Title	1	Identify the report as a systematic review.	Title: "Histopathological Changes Following Bromelain-Based Enzymatic Debridement (NexoBrid®): A Comprehensive Systematic Review of Preclinical and Clinical Evidence"
**ABSTRACT**			
Abstract	2	See the PRISMA 2020 for Abstracts checklist.	Abstract: Unstructured abstract with background, methods, results, and conclusions in flowing prose (Burns & Trauma format)
**INTRODUCTION**			
Rationale	3	Describe the rationale for the review in the context of existing knowledge.	Background section, paragraphs 1-4: burn management burden, surgical debridement limitations (41.2% viable tissue sacrifice), NexoBrid mechanism, histological evidence gap
Objectives	4	Provide an explicit statement of the objective(s) or question(s) the review addresses.	Background section, final paragraph: Five objectives - (1) identify studies, (2) appraise quality, (3) synthesise findings, (4) compare preclinical/clinical evidence, (5) identify research priorities
**METHODS**			
Eligibility criteria	5	Specify the inclusion and exclusion criteria for the review and how studies were grouped for the syntheses.	Review section, Eligibility criteria: PICO criteria for preclinical and clinical arms; Exclusion criteria
Information sources	6	Specify all databases, registers, websites, organisations, reference lists and other sources searched or consulted to identify studies. Specify the date when each source was last searched or consulted.	Review section, Information sources: PubMed/MEDLINE, Embase, Cochrane CENTRAL, Web of

PRISMA 2020 Checklist

Section and Topic	Item #	Checklist item	Location where item is reported
			Science, Scopus; ClinicalTrials.gov, EU Clinical Trials Register, WHO ICTRP
Search strategy	7	Present the full search strategies for all databases, registers and websites, including any filters and limits used.	Review section, Search strategy: (NexoBrid OR anacaulase OR Debrase OR bromelain OR ananain) AND (burn OR thermal injury) AND (histology OR histopathology OR biopsy OR microscopy). No language/date restrictions
Selection process	8	Specify the methods used to decide whether a study met the inclusion criteria of the review, including how many reviewers screened each record and each report retrieved, whether they worked independently, and if applicable, details of automation tools used in the process.	Review section, Study selection: Two reviewers independently screened titles/abstracts, then full-text. Disagreements resolved by consensus
Data collection process	9	Specify the methods used to collect data from reports, including how many reviewers collected data from each report, whether they worked independently, any processes for obtaining or confirming data from study investigators, and if applicable, details of automation tools used in the process.	Review section, Data extraction: Data extraction captured study characteristics, population, intervention details, histological methods, outcomes by tissue compartment and timepoint
Data items	10a	List and define all outcomes for which data were sought. Specify whether all results that were compatible with each outcome domain in each study were sought (e.g. for all measures, time points, analyses), and if not, the methods used to decide which results to collect.	Review section, Eligibility criteria (Outcome): Formal tissue biopsy with histological analysis including H&E staining, special stains, or immunohistochemistry
	10b	List and define all other variables for which data were sought (e.g. participant and intervention characteristics, funding sources). Describe any assumptions made about any missing or unclear information.	Review section and Tables 1, 3, 4: Study characteristics, population, intervention details extracted
Study risk of bias assessment	11	Specify the methods used to assess risk of bias in the included studies, including details of the tool(s) used, how many reviewers assessed each study and whether they worked independently, and if applicable, details of automation tools used in the process.	Review section, Quality assessment: SYRCLE Risk of Bias Tool (preclinical); JBI Critical

Section and Topic	Item #	Checklist item	Location where item is reported
			Appraisal Checklists (clinical). Quality: high (≥75%), moderate (50-74%), low (<50%)
Effect measures	12	Specify for each outcome the effect measure(s) (e.g. risk ratio, mean difference) used in the synthesis or presentation of results.	Tables 2, 5: Percentages, means ± SD, P-values (zone of stasis necrosis %, dermal thickness mm, re-epithelialisation time days)
Synthesis methods	13a	Describe the processes used to decide which studies were eligible for each synthesis (e.g. tabulating the study intervention characteristics and comparing against the planned groups for each synthesis (item #5)).	Review section, Data synthesis: Studies grouped into preclinical and clinical arms; narrative synthesis employed
	13b	Describe any methods required to prepare the data for presentation or synthesis, such as handling of missing summary statistics, or data conversions.	Review section, Data synthesis: Narrative synthesis following SWiM guidelines due to anticipated heterogeneity
	13c	Describe any methods used to tabulate or visually display results of individual studies and syntheses.	Tables 1-5: Structured tables for study characteristics, biopsy protocols, quantitative outcomes, tissue compartment comparisons
	13d	Describe any methods used to synthesize results and provide a rationale for the choice(s). If meta-analysis was performed, describe the model(s), method(s) to identify the presence and extent of statistical heterogeneity, and software package(s) used.	Review section, Data synthesis: Narrative synthesis following SWiM guidelines. No meta-analysis performed
	13e	Describe any methods used to explore possible causes of heterogeneity among study results (e.g. subgroup analysis, meta-regression).	Not applicable - narrative synthesis only; heterogeneity addressed qualitatively in Review section (Discussion)
	13f	Describe any sensitivity analyses conducted to assess robustness of the synthesized results.	Not applicable - no sensitivity analyses conducted
Reporting bias assessment	14	Describe any methods used to assess risk of bias due to missing results in a synthesis (arising from reporting biases).	Review section (Limitations): Potential publication bias

PRISMA 2020 Checklist

Section and Topic	Item #	Checklist item	Location where item is reported
			acknowledged
Certainty assessment	15	Describe any methods used to assess certainty (or confidence) in the body of evidence for an outcome.	Review section, Data synthesis: Modified GRADE approach for certainty assessment
**RESULTS**			
Study selection	16a	Describe the results of the search and selection process, from the number of records identified in the search to the number of studies included in the review, ideally using a flow diagram.	Review section, Findings (Study selection) and Figures 1-2: Preclinical: 89→67→25→6 studies. Clinical: 156→118→15→2 studies. PRISMA flow diagrams provided
	16b	Cite studies that might appear to meet the inclusion criteria, but which were excluded, and explain why they were excluded.	Review section, Findings (Preclinical study selection): Excluded studies cited (Hu 2011, Wu 2012 - firearm wounds; Singer 2018 - ischaemic model; Singer 2023 - visual assessment only)
Study characteristics	17	Cite each included study and present its characteristics.	Tables 1, 3: All 8 studies cited with characteristics (Rowan 1990, Orgill 1996, Singer 2010a/b, Singer 2011, Rosenberg 2012, Di Lonardo 2018, Miura 2025)
Risk of bias in studies	18	Present assessments of risk of bias for each included study.	Review section, Findings (Quality assessment): SYRCLE assessment (moderate quality; Singer 2010a highest); JBI assessment (Di Lonardo 75%, Miura 62.5%)
Results of individual studies	19	For all outcomes, present, for each study: (a) summary statistics for each group (where appropriate) and (b) an effect estimate and its precision (e.g. confidence/credible interval), ideally using structured tables or plots.	Table 2: Quantitative outcomes with means ± SD and P-values. Table 5: Qualitative comparisons by tissue compartment
Results of	20a	For each synthesis, briefly summarise the characteristics and risk of bias among contributing studies.	Review section, Findings (Preclinical and Clinical

Section and Topic	Item #	Checklist item	Location where item is reported
syntheses			evidence): Study characteristics and quality assessments summarised
	20b	Present results of all statistical syntheses conducted. If meta-analysis was done, present for each the summary estimate and its precision (e.g. confidence/credible interval) and measures of statistical heterogeneity. If comparing groups, describe the direction of the effect.	Not applicable - narrative synthesis only. Individual study statistics in Table 2
	20c	Present results of all investigations of possible causes of heterogeneity among study results.	Review section (Discussion): Qualitative discussion of consistency across domains (dermal preservation, selectivity, zone of stasis protection)
	20d	Present results of all sensitivity analyses conducted to assess the robustness of the synthesized results.	Not applicable - no sensitivity analyses conducted
Reporting biases	21	Present assessments of risk of bias due to missing results (arising from reporting biases) for each synthesis assessed.	Review section (Limitations): Publication bias acknowledged; search may have missed non-English literature
Certainty of evidence	22	Present assessments of certainty (or confidence) in the body of evidence for each outcome assessed.	Review section (Discussion), Conclusions: Evidence disparity noted - robust preclinical vs limited clinical data (9 patients). Critical translational gap identified
**DISCUSSION**			
Discussion	23a	Provide a general interpretation of the results in the context of other evidence.	Review section (Discussion): Integration of evidence; mechanistic synthesis (collagen selectivity, α2-macroglobulin protection, temperature gradient)
	23b	Discuss any limitations of the evidence included in the review.	Review section (Limitations): Preclinical limitations (species differences, controlled conditions, short follow-up); Clinical limitations (small samples, qualitative analysis)

PRISMA 2020 Checklist

Section and Topic	Item #	Checklist item	Location where item is reported
	23c	Discuss any limitations of the review processes used.	Review section (Limitations): Review limitations (8 studies, preclinical arm not registered, publication bias, possible missed non-English literature)
	23d	Discuss implications of the results for practice, policy, and future research.	Review section (Discussion, Future research): Clinical implications (wound care, graft timing, pseudoeschar management). Five future research priorities
**OTHER INFORMATION**			
Registration and protocol	24a	Provide registration information for the review, including register name and registration number, or state that the review was not registered.	Abstract, Review section (Protocol and registration): PROSPERO CRD420261278507 (clinical arm). Preclinical arm not registered (PROSPERO scope limitation)
	24b	Indicate where the review protocol can be accessed, or state that a protocol was not prepared.	Review section (Protocol and registration): Protocol registered with PROSPERO (CRD420261278507)
	24c	Describe and explain any amendments to information provided at registration or in the protocol.	Not reported
Support	25	Describe sources of financial or non-financial support for the review, and the role of the funders or sponsors in the review.	Declarations: "This research received no specific grant from any funding agency"
Competing interests	26	Declare any competing interests of review authors.	Declarations: "The authors declare no conflicts of interest"
Availability of data, code and other materials	27	Report which of the following are publicly available and where they can be found: template data collection forms; data extracted from included studies; data used for all analyses; analytic code; any other materials used in the review.	Declarations: "All data are available in cited published sources"

From: Page MJ, McKenzie JE, Bossuyt PM, Boutron I, Hoffmann TC, Mulrow CD, et al. The PRISMA 2020 statement: an updated guideline for reporting systematic reviews. BMJ 2021;372:n71. doi: 10.1136/bmj.n71.
This work is licensed under CC BY 4.0. To view a copy of this license, visit https://creativecommons.org/licenses/by/4.0/
